# 2-[(2-Hy­droxy­naphthalen-1-yl)methyl­idene­amino]-5,6,7,8-tetra­hydro-4*H*-cyclo­hepta­[*b*]thio­phene-3-carbonitrile

**DOI:** 10.1107/S160053681300007X

**Published:** 2013-01-09

**Authors:** Abdullah M. Asiri, Muhammad Nadeem Arshad, Tariq R. Sobahi, Ghulam Mustafa

**Affiliations:** a Chemistry Department, Faculty of Science, King Abdulaziz University, PO Box 80203, Jeddah 21589, Saudi Arabia; b Center of Excellence for Advanced Materials Research (CEAMR), Faculty of Science, King Abdulaziz University, PO Box 80203, Jeddah 21589, Saudi Arabia; c Department of Chemistry, University of Gujrat, Gujrat, Pakistan

## Abstract

Two independent mol­ecules, *A* and *B*, comprise the asymmetric unit of the title compound, C_21_H_18_N_2_OS, with the difference in the angle of orientation between the naphthalene ring system and the mean plane of the cyclo­heptyl ring [16.13 (1) in *A* and 11.48 (5)° in *B*], being evident. The cyclo­heptyl ring adopts a distorted chair conformation in each mol­ecule with r.m.s. deviations of 0.2345 (4) (*A*) and 0.2302 (4) Å (*B*). Intra­molecular O—H⋯N hydrogen bonding generates planar six-membered *S*(6) loops with r.m.s. deviations of 0.0099 (1) (*A*) and 0.0286 (1) Å (*B*).

## Related literature

For the synthesis and related structures, see: Asiri *et al.* (2011*a* ▶,*b* ▶). For graph-set notation, see: Bernstein *et al.* (1995 ▶).
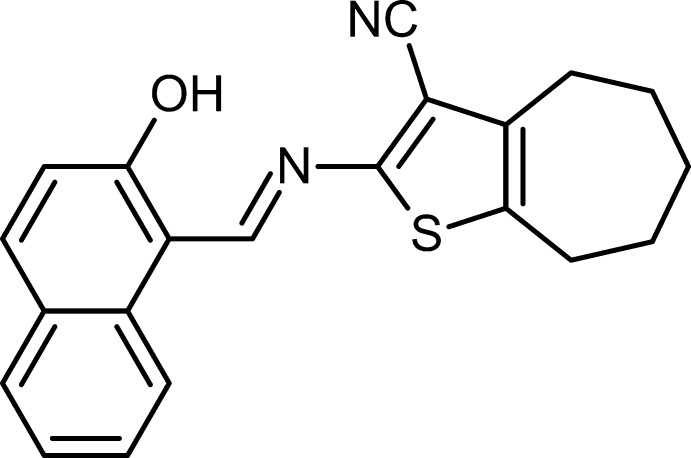


## Experimental

### 

#### Crystal data

C_21_H_18_N_2_OS
*M* *_r_* = 346.43
Orthorhombic, 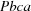

*a* = 13.5472 (2) Å
*b* = 14.4747 (4) Å
*c* = 35.7902 (6) Å
*V* = 7018.2 (2) Å^3^
*Z* = 16
Cu *K*α radiation
μ = 1.71 mm^−1^
*T* = 296 K
0.37 × 0.21 × 0.14 mm

#### Data collection

Agilent SuperNova (Dual, Cu at zero, Atlas, CCD) diffractometer
Absorption correction: multi-scan (*CrysAlis PRO*; Agilent, 2012 ▶) *T*_min_ = 0.858, *T*_max_ = 1.000
28490 measured reflections
7066 independent reflections
5288 reflections with *I* > 2σ(*I*)
*R*_int_ = 0.035

#### Refinement

*R*[*F* ^2^ > 2σ(*F* ^2^)] = 0.054
*wR*(*F* ^2^) = 0.161
*S* = 1.02
7066 reflections
457 parameters
H atoms treated by a mixture of independent and constrained refinement
Δρ_max_ = 0.52 e Å^−3^
Δρ_min_ = −0.25 e Å^−3^

### 

Data collection: *CrysAlis PRO* (Agilent, 2012 ▶); cell refinement: *CrysAlis PRO*; data reduction: *CrysAlis PRO*; program(s) used to solve structure: *SHELXS97* (Sheldrick, 2008 ▶); program(s) used to refine structure: *SHELXL97* (Sheldrick, 2008 ▶); molecular graphics: *PLATON* (Spek, 2009 ▶); software used to prepare material for publication: *WinGX* (Farrugia, 2012 ▶) and *X-SEED* (Barbour, 2001 ▶).

## Supplementary Material

Crystal structure: contains datablock(s) I, global. DOI: 10.1107/S160053681300007X/tk5186sup1.cif

Structure factors: contains datablock(s) I. DOI: 10.1107/S160053681300007X/tk5186Isup2.hkl

Supplementary material file. DOI: 10.1107/S160053681300007X/tk5186Isup3.cml

Additional supplementary materials:  crystallographic information; 3D view; checkCIF report

## Figures and Tables

**Table 1:** Hydrogen-bond geometry (Å, °)

*D*—H⋯*A*	*D*—H	H⋯*A*	*D*⋯*A*	*D*—H⋯*A*
O1—H1*O*⋯N1	0.82 (3)	1.84 (3)	2.578 (2)	150 (3)
O2—H2*O*⋯N3	0.82 (3)	1.84 (3)	2.582 (2)	151 (3)

**Fig. 1.**
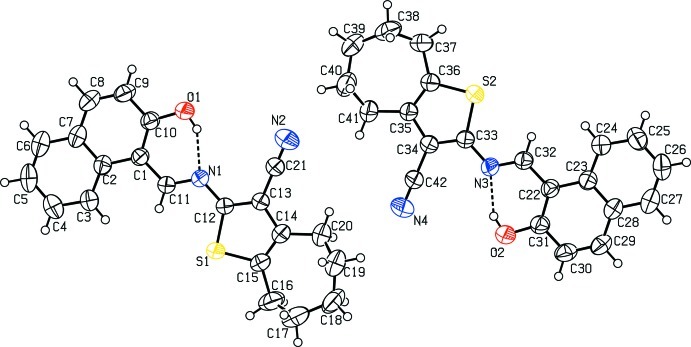
The labelled molecular structures of the two independent molecules comprising the asymmetric unit of (I) with displacement ellipsoids drawn at the 50% probability level. The intramolecular hydrogen bonding shown as dashed lines.

## supplementary crystallographic information

### Comment

In extension of synthesis of Schiff bases containing a thiophene (Asiri *et al.* 2011*a*; 2011*b*) residue, we herein report the crystal structure of title compound.

The title compound (I), Fig. 1, crystallized with two molecules per asymmetric unit. The cycloheptyl ring adopted a chair conformation in each molecule with the r. m. s. deviations being 0.2345 (4) & 0.2302 (4) Å, respectively. The naphthalene ring system is inclined at dihedral angle of 4.97 (4) & 16.13 (1)° with respect to the planes produced from best fitted atoms of thiophene and cycloheptyl rings, respectively in molecule A while the corresponding inclination angles in molecule B are 4.16 (1) & 11.48 (5)°. The thiophene and cycloheptyl rings planes are oriented at dihedral angles of 16.10 (7)° and 15.22 (6)° with respect to each other in molecules A & B. Only intramolecular O—H···N classical hydrogen bonding is observed in both molecules which generates planar six-membered ring motifs *S*(6) (Bernstein *et al.*, 1995) with r. m. s. deviations of 0.0099 (1) & 0.0286 (1) Å from the least-squares planes of member atoms, see Table 1 for details.

### Experimental

The title compound was prepared following literature methods (Asiri *et al.* 2011*a*; 2011*b*) and recrystallized from its methanol solution by slow evaporation.

### Refinement

The C—H H-atoms were positioned with idealized geometry with C—H = 0.93 Å for aromatic-H and C—H = 0.97 Å for methylene groups. H-atoms were refined as riding with *U*_iso_(H) = 1.2*U_eq_*(C). The O—H atoms were refined with *U*_iso_(H) = 1.5*U_eq_*(O).

### Crystal data

**Table:**

C_21_H_18_N_2_OS	*F*(000) = 2912
*M**_r_* = 346.43	*D*_x_ = 1.311 Mg m^−^^3^
Orthorhombic, *P**b**c**a*	Cu *K*α radiation, λ = 1.54184 Å
Hall symbol: -P 2ac 2ab	Cell parameters from 7977 reflections
*a* = 13.5472 (2) Å	θ = 3.3–74.7°
*b* = 14.4747 (4) Å	µ = 1.71 mm^−^^1^
*c* = 35.7902 (6) Å	*T* = 296 K
*V* = 7018.2 (2) Å^3^	Prismatic, dark red
*Z* = 16	0.37 × 0.21 × 0.14 mm

### Data collection

**Table:**

Agilent SuperNova (Dual, Cu at zero, Atlas, CCD) diffractometer	7066 independent reflections
Radiation source: SuperNova (Cu) X-ray Source	5288 reflections with *I* > 2σ(*I*)
Mirror monochromator	*R*_int_ = 0.035
ω scans	θ_max_ = 74.9°, θ_min_ = 4.1°
Absorption correction: multi-scan (*CrysAlis PRO*; Agilent, 2012)	*h* = −16→14
*T*_min_ = 0.858, *T*_max_ = 1.000	*k* = −17→13
28490 measured reflections	*l* = −44→44

### Refinement

**Table:**

Refinement on *F*^2^	Primary atom site location: structure-invariant direct methods
Least-squares matrix: full	Secondary atom site location: difference Fourier map
*R*[*F*^2^ > 2σ(*F*^2^)] = 0.054	Hydrogen site location: inferred from neighbouring sites
*wR*(*F*^2^) = 0.161	H atoms treated by a mixture of independent and constrained refinement
*S* = 1.02	*w* = 1/[σ^2^(*F*_o_^2^) + (0.0753*P*)^2^ + 3.2292*P*] where *P* = (*F*_o_^2^ + 2*F*_c_^2^)/3
7066 reflections	(Δ/σ)_max_ = 0.001
457 parameters	Δρ_max_ = 0.52 e Å^−^^3^
0 restraints	Δρ_min_ = −0.25 e Å^−^^3^

### Special details

**Table:**

Geometry. All esds (except the esd in the dihedral angle between two l.s. planes) are estimated using the full covariance matrix. The cell esds are taken into account individually in the estimation of esds in distances, angles and torsion angles; correlations between esds in cell parameters are only used when they are defined by crystal symmetry. An approximate (isotropic) treatment of cell esds is used for estimating esds involving l.s. planes.
Refinement. Refinement of F^2^ against ALL reflections. The weighted R-factor wR and goodness of fit S are based on F^2^, conventional R-factors R are based on F, with F set to zero for negative F^2^. The threshold expression of F^2^ > 2sigma(F^2^) is used only for calculating R-factors(gt) etc. and is not relevant to the choice of reflections for refinement. R-factors based on F^2^ are statistically about twice as large as those based on F, and R- factors based on ALL data will be even larger.

### Fractional atomic coordinates and isotropic or equivalent isotropic displacement parameters (Å^2^)

**Table:**

	*x*	*y*	*z*	*U*_iso_*/*U*_eq_	
S1	0.24913 (4)	0.21343 (5)	0.314590 (15)	0.05676 (19)	
S2	0.45786 (4)	0.50708 (5)	0.564135 (16)	0.05789 (19)	
O1	0.30203 (12)	0.26884 (15)	0.46051 (5)	0.0657 (5)	
O2	0.51390 (13)	0.57956 (16)	0.70915 (5)	0.0684 (5)	
N1	0.27388 (13)	0.23563 (13)	0.39056 (5)	0.0472 (4)	
N2	0.0201 (2)	0.2458 (3)	0.42460 (7)	0.0989 (10)	
N3	0.48552 (13)	0.54653 (14)	0.63907 (5)	0.0510 (5)	
N4	0.2337 (2)	0.5752 (3)	0.67298 (7)	0.0986 (10)	
C1	0.43541 (16)	0.24092 (16)	0.41754 (6)	0.0459 (5)	
C2	0.54027 (16)	0.23125 (16)	0.41241 (6)	0.0499 (5)	
C3	0.58494 (18)	0.2128 (2)	0.37759 (8)	0.0645 (7)	
H3	0.5456	0.2070	0.3564	0.077*	
C4	0.6851 (2)	0.2034 (2)	0.37445 (10)	0.0812 (9)	
H4	0.7130	0.1908	0.3513	0.097*	
C5	0.7461 (2)	0.2124 (2)	0.40592 (11)	0.0816 (9)	
H5	0.8141	0.2064	0.4035	0.098*	
C6	0.7060 (2)	0.2298 (2)	0.43951 (9)	0.0713 (8)	
H6	0.7470	0.2356	0.4602	0.086*	
C7	0.60312 (17)	0.23961 (17)	0.44399 (7)	0.0557 (6)	
C8	0.56125 (19)	0.25724 (19)	0.47943 (7)	0.0625 (7)	
H8	0.6025	0.2626	0.5001	0.075*	
C9	0.46320 (19)	0.2665 (2)	0.48411 (7)	0.0611 (6)	
H9	0.4377	0.2780	0.5078	0.073*	
C10	0.39935 (17)	0.25896 (17)	0.45341 (6)	0.0511 (5)	
C11	0.36858 (16)	0.23066 (16)	0.38672 (6)	0.0475 (5)	
H11	0.3944	0.2200	0.3630	0.057*	
C12	0.21176 (16)	0.22661 (16)	0.36021 (6)	0.0465 (5)	
C13	0.11097 (16)	0.22964 (19)	0.36215 (6)	0.0531 (6)	
C14	0.06278 (18)	0.2232 (2)	0.32699 (7)	0.0670 (7)	
C15	0.12889 (19)	0.2154 (2)	0.29865 (7)	0.0673 (7)	
C16	0.1107 (3)	0.2146 (3)	0.25696 (8)	0.0941 (11)	
H16A	0.0941	0.2768	0.2492	0.113*	
H16B	0.1718	0.1978	0.2446	0.113*	
C17	0.0333 (3)	0.1525 (3)	0.24402 (9)	0.1011 (12)	
H17A	0.0490	0.0907	0.2527	0.121*	
H17B	0.0352	0.1512	0.2169	0.121*	
C18	−0.0719 (3)	0.1746 (3)	0.25595 (9)	0.0967 (11)	
H18A	−0.0866	0.2377	0.2487	0.116*	
H18B	−0.1165	0.1346	0.2422	0.116*	
C19	−0.0935 (2)	0.1643 (3)	0.29717 (11)	0.1069 (13)	
H19A	−0.1644	0.1677	0.3006	0.128*	
H19B	−0.0728	0.1030	0.3048	0.128*	
C20	−0.0477 (2)	0.2311 (3)	0.32213 (10)	0.1028 (13)	
H20A	−0.0783	0.2254	0.3465	0.123*	
H20B	−0.0624	0.2926	0.3129	0.123*	
C21	0.06072 (18)	0.2395 (2)	0.39699 (7)	0.0646 (7)	
C22	0.64645 (16)	0.54730 (16)	0.66648 (6)	0.0489 (5)	
C23	0.75161 (17)	0.53553 (17)	0.66162 (6)	0.0508 (5)	
C24	0.79674 (19)	0.5194 (2)	0.62665 (7)	0.0635 (7)	
H24	0.7578	0.5162	0.6053	0.076*	
C25	0.8967 (2)	0.5085 (2)	0.62369 (9)	0.0763 (8)	
H25	0.9245	0.4973	0.6004	0.092*	
C26	0.9576 (2)	0.5139 (2)	0.65491 (10)	0.0767 (8)	
H26	1.0255	0.5066	0.6525	0.092*	
C27	0.9170 (2)	0.5298 (2)	0.68894 (9)	0.0699 (8)	
H27	0.9577	0.5332	0.7098	0.084*	
C28	0.81442 (18)	0.54127 (18)	0.69330 (7)	0.0557 (6)	
C29	0.77185 (19)	0.55757 (19)	0.72897 (7)	0.0621 (7)	
H29	0.8127	0.5596	0.7498	0.075*	
C30	0.6739 (2)	0.57016 (19)	0.73347 (7)	0.0622 (6)	
H30	0.6483	0.5815	0.7571	0.075*	
C31	0.61033 (18)	0.56608 (18)	0.70233 (6)	0.0535 (6)	
C32	0.57975 (16)	0.53732 (17)	0.63555 (6)	0.0508 (5)	
H32	0.6055	0.5237	0.6121	0.061*	
C33	0.42264 (16)	0.53346 (17)	0.60903 (6)	0.0489 (5)	
C34	0.32167 (16)	0.54044 (17)	0.61095 (6)	0.0500 (5)	
C35	0.27183 (17)	0.52450 (18)	0.57632 (6)	0.0526 (6)	
C36	0.33707 (18)	0.50529 (19)	0.54855 (7)	0.0563 (6)	
C37	0.3190 (2)	0.4775 (3)	0.50862 (7)	0.0785 (9)	
H37A	0.3811	0.4810	0.4952	0.094*	
H37B	0.2980	0.4134	0.5083	0.094*	
C38	0.2441 (3)	0.5336 (3)	0.48794 (8)	0.0933 (11)	
H38A	0.2462	0.5159	0.4618	0.112*	
H38B	0.2633	0.5981	0.4894	0.112*	
C39	0.1380 (2)	0.5250 (3)	0.50151 (9)	0.0816 (9)	
H39A	0.0947	0.5523	0.4830	0.098*	
H39B	0.1215	0.4600	0.5034	0.098*	
C40	0.1183 (2)	0.5701 (2)	0.53854 (8)	0.0778 (8)	
H40A	0.1429	0.6329	0.5373	0.093*	
H40B	0.0473	0.5737	0.5418	0.093*	
C41	0.16098 (18)	0.5257 (2)	0.57281 (8)	0.0686 (7)	
H41A	0.1378	0.4623	0.5738	0.082*	
H41B	0.1344	0.5572	0.5945	0.082*	
C42	0.27270 (18)	0.5608 (2)	0.64544 (7)	0.0624 (7)	
H1O	0.272 (3)	0.261 (2)	0.4409 (9)	0.094*	
H2O	0.486 (3)	0.577 (3)	0.6889 (9)	0.094*	

### Atomic displacement parameters (Å^2^)

**Table:**

	*U*^11^	*U*^22^	*U*^33^	*U*^12^	*U*^13^	*U*^23^
S1	0.0477 (3)	0.0840 (5)	0.0386 (3)	0.0013 (3)	0.0038 (2)	−0.0040 (3)
S2	0.0453 (3)	0.0822 (5)	0.0461 (3)	0.0019 (3)	0.0062 (2)	−0.0034 (3)
O1	0.0496 (10)	0.1030 (15)	0.0445 (9)	−0.0027 (9)	0.0032 (7)	−0.0078 (9)
O2	0.0559 (10)	0.1014 (15)	0.0480 (9)	0.0012 (10)	0.0052 (8)	−0.0017 (10)
N1	0.0428 (9)	0.0580 (11)	0.0408 (9)	0.0009 (8)	−0.0020 (7)	−0.0018 (8)
N2	0.0766 (17)	0.156 (3)	0.0644 (15)	0.0023 (18)	0.0201 (14)	−0.0130 (17)
N3	0.0453 (10)	0.0644 (13)	0.0432 (9)	0.0002 (9)	−0.0012 (8)	0.0046 (9)
N4	0.0770 (17)	0.167 (3)	0.0524 (13)	0.0185 (18)	0.0161 (12)	−0.0012 (17)
C1	0.0453 (11)	0.0495 (13)	0.0430 (11)	−0.0007 (9)	−0.0035 (9)	0.0009 (9)
C2	0.0451 (11)	0.0516 (13)	0.0528 (12)	−0.0020 (10)	−0.0029 (10)	0.0047 (11)
C3	0.0458 (13)	0.0838 (19)	0.0640 (15)	−0.0004 (12)	0.0018 (11)	−0.0035 (14)
C4	0.0553 (15)	0.101 (2)	0.087 (2)	0.0032 (15)	0.0154 (15)	−0.0028 (18)
C5	0.0381 (13)	0.091 (2)	0.116 (3)	0.0000 (13)	−0.0038 (15)	0.005 (2)
C6	0.0500 (14)	0.0738 (19)	0.090 (2)	−0.0047 (13)	−0.0174 (14)	0.0081 (16)
C7	0.0485 (12)	0.0513 (14)	0.0673 (15)	−0.0053 (10)	−0.0132 (11)	0.0071 (12)
C8	0.0644 (16)	0.0688 (17)	0.0543 (13)	−0.0093 (13)	−0.0190 (12)	0.0073 (12)
C9	0.0662 (15)	0.0746 (18)	0.0424 (11)	−0.0086 (13)	−0.0081 (11)	0.0022 (12)
C10	0.0504 (12)	0.0585 (14)	0.0444 (11)	−0.0042 (10)	−0.0018 (9)	0.0021 (10)
C11	0.0442 (11)	0.0569 (14)	0.0413 (10)	0.0013 (9)	0.0005 (9)	−0.0016 (10)
C12	0.0447 (11)	0.0569 (14)	0.0378 (10)	0.0019 (9)	−0.0003 (8)	−0.0013 (10)
C13	0.0435 (11)	0.0726 (16)	0.0432 (11)	0.0011 (10)	0.0010 (9)	−0.0052 (11)
C14	0.0486 (13)	0.102 (2)	0.0505 (13)	0.0009 (13)	−0.0074 (11)	−0.0089 (14)
C15	0.0577 (14)	0.101 (2)	0.0428 (12)	−0.0007 (14)	−0.0074 (11)	−0.0055 (13)
C16	0.091 (2)	0.147 (3)	0.0446 (14)	−0.017 (2)	−0.0099 (14)	−0.0047 (18)
C17	0.109 (3)	0.137 (3)	0.0577 (17)	−0.005 (2)	−0.0232 (17)	−0.011 (2)
C18	0.086 (2)	0.121 (3)	0.084 (2)	0.008 (2)	−0.0430 (18)	−0.014 (2)
C19	0.0621 (19)	0.157 (4)	0.102 (3)	0.001 (2)	−0.0229 (18)	−0.015 (3)
C20	0.0498 (16)	0.177 (4)	0.082 (2)	0.008 (2)	−0.0145 (15)	−0.027 (2)
C21	0.0475 (13)	0.097 (2)	0.0490 (13)	0.0010 (13)	0.0024 (11)	−0.0069 (13)
C22	0.0480 (12)	0.0530 (13)	0.0457 (11)	−0.0017 (10)	−0.0027 (9)	0.0044 (10)
C23	0.0476 (12)	0.0535 (13)	0.0511 (12)	−0.0008 (10)	−0.0028 (10)	0.0020 (11)
C24	0.0524 (14)	0.0793 (18)	0.0587 (14)	0.0019 (12)	−0.0005 (11)	−0.0054 (13)
C25	0.0558 (15)	0.091 (2)	0.0816 (19)	0.0042 (14)	0.0106 (14)	−0.0116 (17)
C26	0.0463 (14)	0.083 (2)	0.101 (2)	0.0052 (13)	−0.0027 (15)	−0.0004 (18)
C27	0.0543 (15)	0.0727 (18)	0.0826 (19)	−0.0016 (13)	−0.0186 (14)	0.0064 (15)
C28	0.0542 (13)	0.0544 (14)	0.0584 (13)	−0.0011 (11)	−0.0112 (11)	0.0046 (11)
C29	0.0654 (16)	0.0699 (17)	0.0512 (13)	−0.0060 (13)	−0.0144 (11)	0.0074 (12)
C30	0.0710 (16)	0.0732 (17)	0.0424 (11)	−0.0082 (13)	−0.0028 (11)	0.0042 (12)
C31	0.0523 (13)	0.0619 (15)	0.0463 (12)	−0.0036 (11)	0.0012 (10)	0.0037 (11)
C32	0.0486 (12)	0.0598 (14)	0.0441 (11)	−0.0012 (10)	0.0005 (9)	0.0043 (10)
C33	0.0463 (12)	0.0581 (14)	0.0422 (11)	0.0008 (10)	0.0015 (9)	0.0038 (10)
C34	0.0461 (11)	0.0604 (14)	0.0434 (11)	−0.0004 (10)	0.0031 (9)	0.0021 (10)
C35	0.0450 (12)	0.0667 (15)	0.0460 (11)	−0.0010 (10)	−0.0003 (9)	0.0011 (11)
C36	0.0508 (13)	0.0720 (16)	0.0462 (12)	−0.0018 (11)	0.0014 (10)	−0.0043 (11)
C37	0.0730 (18)	0.113 (3)	0.0492 (14)	−0.0037 (17)	0.0027 (13)	−0.0168 (15)
C38	0.102 (2)	0.130 (3)	0.0482 (15)	−0.006 (2)	−0.0155 (16)	−0.0017 (17)
C39	0.0742 (19)	0.101 (2)	0.0699 (18)	−0.0038 (17)	−0.0273 (15)	−0.0072 (17)
C40	0.0606 (16)	0.090 (2)	0.083 (2)	0.0038 (15)	−0.0208 (14)	−0.0045 (17)
C41	0.0473 (13)	0.095 (2)	0.0639 (15)	0.0006 (13)	−0.0045 (12)	0.0006 (15)
C42	0.0485 (13)	0.093 (2)	0.0461 (12)	0.0072 (13)	0.0028 (10)	0.0027 (13)

### Geometric parameters (Å, º)

**Table:**

S1—C12	1.720 (2)	C18—H18A	0.9700
S1—C15	1.726 (3)	C18—H18B	0.9700
S2—C33	1.719 (2)	C19—C20	1.456 (5)
S2—C36	1.729 (2)	C19—H19A	0.9700
O1—C10	1.350 (3)	C19—H19B	0.9700
O1—H1O	0.82 (3)	C20—H20A	0.9700
O2—C31	1.343 (3)	C20—H20B	0.9700
O2—H2O	0.82 (3)	C22—C31	1.400 (3)
N1—C11	1.292 (3)	C22—C32	1.436 (3)
N1—C12	1.380 (3)	C22—C23	1.445 (3)
N2—C21	1.135 (3)	C23—C24	1.412 (3)
N3—C32	1.290 (3)	C23—C28	1.420 (3)
N3—C33	1.385 (3)	C24—C25	1.368 (4)
N4—C42	1.137 (3)	C24—H24	0.9300
C1—C10	1.398 (3)	C25—C26	1.391 (4)
C1—C11	1.435 (3)	C25—H25	0.9300
C1—C2	1.439 (3)	C26—C27	1.356 (4)
C2—C3	1.411 (3)	C26—H26	0.9300
C2—C7	1.420 (3)	C27—C28	1.408 (4)
C3—C4	1.368 (4)	C27—H27	0.9300
C3—H3	0.9300	C28—C29	1.420 (4)
C4—C5	1.403 (4)	C29—C30	1.349 (4)
C4—H4	0.9300	C29—H29	0.9300
C5—C6	1.343 (4)	C30—C31	1.410 (3)
C5—H5	0.9300	C30—H30	0.9300
C6—C7	1.410 (4)	C32—H32	0.9300
C6—H6	0.9300	C33—C34	1.373 (3)
C7—C8	1.413 (4)	C34—C35	1.430 (3)
C8—C9	1.346 (4)	C34—C42	1.432 (3)
C8—H8	0.9300	C35—C36	1.359 (3)
C9—C10	1.403 (3)	C35—C41	1.507 (3)
C9—H9	0.9300	C36—C37	1.505 (3)
C11—H11	0.9300	C37—C38	1.495 (5)
C12—C13	1.368 (3)	C37—H37A	0.9700
C13—C14	1.421 (3)	C37—H37B	0.9700
C13—C21	1.428 (3)	C38—C39	1.522 (5)
C14—C15	1.358 (4)	C38—H38A	0.9700
C14—C20	1.511 (4)	C38—H38B	0.9700
C15—C16	1.513 (3)	C39—C40	1.501 (4)
C16—C17	1.457 (5)	C39—H39A	0.9700
C16—H16A	0.9700	C39—H39B	0.9700
C16—H16B	0.9700	C40—C41	1.500 (4)
C17—C18	1.522 (5)	C40—H40A	0.9700
C17—H17A	0.9700	C40—H40B	0.9700
C17—H17B	0.9700	C41—H41A	0.9700
C18—C19	1.511 (5)	C41—H41B	0.9700
			
C12—S1—C15	91.95 (11)	C14—C20—H20B	108.2
C33—S2—C36	92.41 (11)	H20A—C20—H20B	107.4
C10—O1—H1O	108 (2)	N2—C21—C13	178.8 (4)
C31—O2—H2O	106 (2)	C31—C22—C32	120.4 (2)
C11—N1—C12	121.09 (19)	C31—C22—C23	118.5 (2)
C32—N3—C33	121.3 (2)	C32—C22—C23	121.0 (2)
C10—C1—C11	120.3 (2)	C24—C23—C28	117.3 (2)
C10—C1—C2	118.7 (2)	C24—C23—C22	123.5 (2)
C11—C1—C2	121.0 (2)	C28—C23—C22	119.2 (2)
C3—C2—C7	117.5 (2)	C25—C24—C23	121.1 (3)
C3—C2—C1	123.7 (2)	C25—C24—H24	119.5
C7—C2—C1	118.8 (2)	C23—C24—H24	119.5
C4—C3—C2	121.1 (3)	C24—C25—C26	121.2 (3)
C4—C3—H3	119.4	C24—C25—H25	119.4
C2—C3—H3	119.4	C26—C25—H25	119.4
C3—C4—C5	120.6 (3)	C27—C26—C25	119.3 (3)
C3—C4—H4	119.7	C27—C26—H26	120.3
C5—C4—H4	119.7	C25—C26—H26	120.3
C6—C5—C4	119.9 (3)	C26—C27—C28	121.4 (3)
C6—C5—H5	120.0	C26—C27—H27	119.3
C4—C5—H5	120.0	C28—C27—H27	119.3
C5—C6—C7	121.3 (3)	C27—C28—C23	119.7 (2)
C5—C6—H6	119.3	C27—C28—C29	121.3 (2)
C7—C6—H6	119.3	C23—C28—C29	118.9 (2)
C6—C7—C8	121.1 (2)	C30—C29—C28	122.0 (2)
C6—C7—C2	119.6 (3)	C30—C29—H29	119.0
C8—C7—C2	119.3 (2)	C28—C29—H29	119.0
C9—C8—C7	121.8 (2)	C29—C30—C31	120.0 (2)
C9—C8—H8	119.1	C29—C30—H30	120.0
C7—C8—H8	119.1	C31—C30—H30	120.0
C8—C9—C10	120.2 (2)	O2—C31—C22	122.3 (2)
C8—C9—H9	119.9	O2—C31—C30	116.4 (2)
C10—C9—H9	119.9	C22—C31—C30	121.3 (2)
O1—C10—C1	122.3 (2)	N3—C32—C22	122.5 (2)
O1—C10—C9	116.5 (2)	N3—C32—H32	118.8
C1—C10—C9	121.2 (2)	C22—C32—H32	118.8
N1—C11—C1	122.6 (2)	C34—C33—N3	124.3 (2)
N1—C11—H11	118.7	C34—C33—S2	109.85 (17)
C1—C11—H11	118.7	N3—C33—S2	125.82 (17)
C13—C12—N1	124.44 (19)	C33—C34—C35	114.5 (2)
C13—C12—S1	110.22 (16)	C33—C34—C42	121.3 (2)
N1—C12—S1	125.31 (16)	C35—C34—C42	124.2 (2)
C12—C13—C14	114.3 (2)	C36—C35—C34	111.1 (2)
C12—C13—C21	121.6 (2)	C36—C35—C41	126.1 (2)
C14—C13—C21	124.1 (2)	C34—C35—C41	122.7 (2)
C15—C14—C13	111.3 (2)	C35—C36—C37	130.1 (2)
C15—C14—C20	125.0 (3)	C35—C36—S2	112.13 (18)
C13—C14—C20	123.5 (2)	C37—C36—S2	117.67 (19)
C14—C15—C16	129.1 (3)	C38—C37—C36	115.8 (3)
C14—C15—S1	112.15 (18)	C38—C37—H37A	108.3
C16—C15—S1	118.6 (2)	C36—C37—H37A	108.3
C17—C16—C15	115.8 (3)	C38—C37—H37B	108.3
C17—C16—H16A	108.3	C36—C37—H37B	108.3
C15—C16—H16A	108.3	H37A—C37—H37B	107.4
C17—C16—H16B	108.3	C37—C38—C39	116.0 (3)
C15—C16—H16B	108.3	C37—C38—H38A	108.3
H16A—C16—H16B	107.4	C39—C38—H38A	108.3
C16—C17—C18	117.1 (3)	C37—C38—H38B	108.3
C16—C17—H17A	108.0	C39—C38—H38B	108.3
C18—C17—H17A	108.0	H38A—C38—H38B	107.4
C16—C17—H17B	108.0	C40—C39—C38	114.5 (2)
C18—C17—H17B	108.0	C40—C39—H39A	108.6
H17A—C17—H17B	107.3	C38—C39—H39A	108.6
C19—C18—C17	115.7 (3)	C40—C39—H39B	108.6
C19—C18—H18A	108.3	C38—C39—H39B	108.6
C17—C18—H18A	108.3	H39A—C39—H39B	107.6
C19—C18—H18B	108.3	C41—C40—C39	117.8 (3)
C17—C18—H18B	108.3	C41—C40—H40A	107.8
H18A—C18—H18B	107.4	C39—C40—H40A	107.8
C20—C19—C18	116.8 (4)	C41—C40—H40B	107.8
C20—C19—H19A	108.1	C39—C40—H40B	107.8
C18—C19—H19A	108.1	H40A—C40—H40B	107.2
C20—C19—H19B	108.1	C40—C41—C35	117.2 (2)
C18—C19—H19B	108.1	C40—C41—H41A	108.0
H19A—C19—H19B	107.3	C35—C41—H41A	108.0
C19—C20—C14	116.3 (3)	C40—C41—H41B	108.0
C19—C20—H20A	108.2	C35—C41—H41B	108.0
C14—C20—H20A	108.2	H41A—C41—H41B	107.2
C19—C20—H20B	108.2	N4—C42—C34	178.7 (4)
			
C10—C1—C2—C3	179.8 (2)	C31—C22—C23—C24	−177.8 (2)
C11—C1—C2—C3	1.0 (4)	C32—C22—C23—C24	4.0 (4)
C10—C1—C2—C7	0.5 (3)	C31—C22—C23—C28	1.4 (4)
C11—C1—C2—C7	−178.3 (2)	C32—C22—C23—C28	−176.8 (2)
C7—C2—C3—C4	0.0 (4)	C28—C23—C24—C25	1.0 (4)
C1—C2—C3—C4	−179.3 (3)	C22—C23—C24—C25	−179.8 (3)
C2—C3—C4—C5	−0.4 (5)	C23—C24—C25—C26	−0.7 (5)
C3—C4—C5—C6	0.5 (5)	C24—C25—C26—C27	0.3 (5)
C4—C5—C6—C7	−0.2 (5)	C25—C26—C27—C28	−0.2 (5)
C5—C6—C7—C8	179.5 (3)	C26—C27—C28—C23	0.4 (4)
C5—C6—C7—C2	−0.2 (4)	C26—C27—C28—C29	179.7 (3)
C3—C2—C7—C6	0.3 (4)	C24—C23—C28—C27	−0.8 (4)
C1—C2—C7—C6	179.7 (2)	C22—C23—C28—C27	179.9 (2)
C3—C2—C7—C8	−179.5 (2)	C24—C23—C28—C29	179.9 (2)
C1—C2—C7—C8	−0.1 (4)	C22—C23—C28—C29	0.6 (4)
C6—C7—C8—C9	−179.9 (3)	C27—C28—C29—C30	178.9 (3)
C2—C7—C8—C9	−0.1 (4)	C23—C28—C29—C30	−1.8 (4)
C7—C8—C9—C10	−0.1 (4)	C28—C29—C30—C31	0.9 (4)
C11—C1—C10—O1	−1.1 (4)	C32—C22—C31—O2	−3.0 (4)
C2—C1—C10—O1	−179.9 (2)	C23—C22—C31—O2	178.8 (2)
C11—C1—C10—C9	178.1 (2)	C32—C22—C31—C30	175.8 (2)
C2—C1—C10—C9	−0.8 (4)	C23—C22—C31—C30	−2.4 (4)
C8—C9—C10—O1	179.8 (2)	C29—C30—C31—O2	−179.9 (2)
C8—C9—C10—C1	0.6 (4)	C29—C30—C31—C22	1.3 (4)
C12—N1—C11—C1	179.2 (2)	C33—N3—C32—C22	−177.8 (2)
C10—C1—C11—N1	−1.4 (4)	C31—C22—C32—N3	1.2 (4)
C2—C1—C11—N1	177.5 (2)	C23—C22—C32—N3	179.3 (2)
C11—N1—C12—C13	178.9 (2)	C32—N3—C33—C34	178.7 (2)
C11—N1—C12—S1	−3.1 (3)	C32—N3—C33—S2	−1.7 (3)
C15—S1—C12—C13	1.5 (2)	C36—S2—C33—C34	−0.2 (2)
C15—S1—C12—N1	−176.7 (2)	C36—S2—C33—N3	−179.8 (2)
N1—C12—C13—C14	177.1 (2)	N3—C33—C34—C35	179.7 (2)
S1—C12—C13—C14	−1.1 (3)	S2—C33—C34—C35	0.1 (3)
N1—C12—C13—C21	−2.2 (4)	N3—C33—C34—C42	−1.2 (4)
S1—C12—C13—C21	179.5 (2)	S2—C33—C34—C42	179.2 (2)
C12—C13—C14—C15	−0.1 (4)	C33—C34—C35—C36	0.2 (3)
C21—C13—C14—C15	179.3 (3)	C42—C34—C35—C36	−178.9 (3)
C12—C13—C14—C20	−176.0 (3)	C33—C34—C35—C41	177.8 (2)
C21—C13—C14—C20	3.4 (5)	C42—C34—C35—C41	−1.3 (4)
C13—C14—C15—C16	−174.5 (3)	C34—C35—C36—C37	175.1 (3)
C20—C14—C15—C16	1.3 (6)	C41—C35—C36—C37	−2.5 (5)
C13—C14—C15—S1	1.3 (4)	C34—C35—C36—S2	−0.3 (3)
C20—C14—C15—S1	177.1 (3)	C41—C35—C36—S2	−177.9 (2)
C12—S1—C15—C14	−1.6 (3)	C33—S2—C36—C35	0.3 (2)
C12—S1—C15—C16	174.6 (3)	C33—S2—C36—C37	−175.7 (2)
C14—C15—C16—C17	−48.8 (6)	C35—C36—C37—C38	47.0 (5)
S1—C15—C16—C17	135.7 (3)	S2—C36—C37—C38	−137.9 (3)
C15—C16—C17—C18	66.1 (5)	C36—C37—C38—C39	−66.9 (4)
C16—C17—C18—C19	−67.5 (5)	C37—C38—C39—C40	71.3 (4)
C17—C18—C19—C20	69.4 (5)	C38—C39—C40—C41	−71.0 (4)
C18—C19—C20—C14	−70.2 (5)	C39—C40—C41—C35	65.8 (4)
C15—C14—C20—C19	48.6 (6)	C36—C35—C41—C40	−42.6 (4)
C13—C14—C20—C19	−136.1 (4)	C34—C35—C41—C40	140.1 (3)

### Hydrogen-bond geometry (Å, º)

**Table:**

*D*—H···*A*	*D*—H	H···*A*	*D*···*A*	*D*—H···*A*
O1—H1*O*···N1	0.82 (3)	1.84 (3)	2.578 (2)	150 (3)
O2—H2*O*···N3	0.82 (3)	1.84 (3)	2.582 (2)	151 (3)
